# Integrated metabolomic and transcriptomic analysis of anthocyanin accumulation mechanisms in maize kernels of different colors

**DOI:** 10.3389/fgene.2026.1797093

**Published:** 2026-06-01

**Authors:** Chao Jiang, Hanbo Shi, Linan Yan, Wenxu Yin, Hui Li, Haibo Lu, Haichao Zhao, Songtao Liu, Shuo Wang, Dong Wei, Zhihong Huang

**Affiliations:** 1 Hebei Key Laboratory of Quality and Safety Analysis-Testing for Agro-Products and Food, Hebei North University, Zhangjiakou, China; 2 College of Agriculture, Forestry and Technology, Hebei North University, Zhangjiakou, China; 3 School of Medicine, Nankai University, Tianjin, China

**Keywords:** anthocyanins, maize, metabolomics, structural genes, transcriptomics

## Abstract

**Introduction:**

Anthocyanins are important natural pigments and bioactive compounds in colored maize kernels. Although colored fresh-eating maize has attracted increasing attention because of its nutritional and functional value, the molecular basis underlying anthocyanin accumulation in maize kernels with different colors remains incompletely understood.

**Methods:**

In this study, four fresh-eating maize cultivars with contrasting kernel colors, including white (WN 2000), yellow (HN), multicolored (CN1), and black (HTN188), were used to investigate the mechanism of anthocyanin accumulation. Total anthocyanin content was measured across developmental stages, and integrated metabolomic and transcriptomic analyses were performed to identify anthocyanin-related metabolites, differentially expressed genes, and candidate regulatory factors associated with kernel pigmentation.

**Results:**

Metabolomic profiling identified 49 anthocyanin-related metabolites/features, with marked enrichment of cyanidin-, peonidin-, and pelargonidin-related compounds in the black maize cultivar HTN188. Transcriptomic analysis identified 12,557 differentially expressed genes among the four cultivars, which were mainly enriched in phenylpropanoid biosynthesis, flavonoid biosynthesis, glutathione metabolism, fructose and mannose metabolism, and glyoxylate and dicarboxylate metabolism pathways. Several anthocyanin biosynthetic genes and regulatory transcription factor candidates were upregulated in anthocyanin-rich kernels, including DFR (Zm00001d011438), MYB (Zm00001d003052), bHLH (Zm00001d015990), and NAC (Zm00001d012508). In particular, the expression of DFR (Zm00001d011438) was strongly positively correlated with total anthocyanin content. Weighted gene co-expression network analysis further identified gene modules and candidate hub regulators associated with anthocyanin accumulation.

**Discussion:**

These findings provide a comparative multi-omics perspective on anthocyanin accumulation in fresh-eating maize kernels of different colors. The results suggest that the high anthocyanin accumulation in black maize is associated with the coordinated enrichment of anthocyanin-related metabolites and the upregulation of key structural genes and transcription factors. This study provides candidate genes and regulatory information for future functional studies and anthocyanin-oriented breeding in fresh-eating maize.

## 1 Introduction

Maize (*Zea mays L.*) is one of the three major staple crops worldwide, along with rice and wheat. It belongs to the genus Zea of the family Poaceae. In addition to its role as both food and feed, maize also has applications in the pharmaceutical and cosmetic industries (Colombo et al., 2021). Compared with conventional maize, sweet maize contains a higher amylopectin content, which contributes to enhanced viscosity, adhesiveness, and digestibility. Furthermore, sweet maize is not only rich in protein and starch but also represents an important cereal source of anthocyanins (Singh et al., 2006; Cuevas Montilla et al., 2011). In response to increasing health consciousness in modern society, foods with high bioactivity have gained substantial attention. Colored sweet maize stands out due to its elevated levels of anthocyanins and phenolic compounds, drawing growing interest from researchers and consumers alike (Petroni et al., 2014; Lao and Giusti, 2017; Guo et al., 2021).

Anthocyanins are a class of water-soluble flavonoid polyphenols that are widely distributed across plant species (Rhowell et al., 2014). Structurally, anthocyanins are glycosides, and their aglycone forms are referred to as anthocyanidins. With the exception of algae, anthocyanin synthesis has been observed in virtually all higher plant taxa. They are among the primary pigments responsible for the coloration of fruits and petals. The perceived hue of anthocyanin pigments is strongly influenced by vacuolar pH, which shifts the equilibrium among different molecular forms and thus affects color expression. However, hue and stability are also modulated by structural features and supramolecular interactions, notably co-pigmentation with other phenolics/flavones and complexation with metal ions, which can cause bathochromic and/or hyperchromic shifts and contribute to blue–purple coloration (He and Giusti, 2010; Tanaka and Brugliera, 2009). The intensity of the color correlates with the concentration of anthocyanins present (Cooper-Driver, 2001; Steyn, 2008). These compounds are commonly found in floral tissues, fruits, and the epidermal layers of stems and leaves.

Currently, six anthocyanidins are most commonly identified: delphinidin (Dp), cyanidin (Cy), petunidin (Pt), pelargonidin (Pg), peonidin (Pn), and malvidin (Mv) (Kong et al., 2003). As natural pigments, anthocyanins significantly contribute to plant antioxidant capabilities and stress resistance. In human health, they are associated with various physiological benefits, including antioxidant activity, anti-aging effects, tumor suppression, cardiovascular protection, hepatic function enhancement, and vision support (Yousuf et al., 2016). Compared with fruits, pigmented maize kernels represent a dehydrated and more cost-effective anthocyanin source for industrial extraction (Haggard et al., 2018). At the molecular level, anthocyanin accumulation is largely controlled transcriptionally through coordinated regulation of structural genes in the phenylpropanoid/flavonoid pathway (e.g., PAL, CHS, CHI, F3H, DFR, ANS/LDOX, and UFGT) (Hichri et al., 2011; Jaakola, 2013). A conserved regulatory framework involves the MYB–bHLH–WD40 (MBW) complex, in which R2R3-MYB and bHLH transcription factors interact with WD40 proteins to activate (or repress) anthocyanin pathway genes in a tissue- and developmental stage-dependent manner (Gonzalez et al., 2008; Hichri et al., 2011; Zhang et al., 2018). In maize, combinations of MYB- and bHLH-type regulators, together with WD40 cofactors, are associated with kernel pigmentation by modulating the expression of these structural genes and downstream processes such as anthocyanin transport and vacuolar sequestration. In addition to the core MBW module, other TF families (e.g., WRKY and NAC) have been reported to influence anthocyanin-associated gene expression, likely by acting upstream of, or in parallel with, the MBW regulatory module (Lai et al., 2015; Gou et al., 2011; Hou et al., 2022; Fan et al., 2017).

In recent years, advances in sequencing technologies and the availability of genomic data for various model plants have enabled high-throughput omics approaches to become powerful tools in elucidating anthocyanin biosynthesis pathways. Among these, transcriptomics and metabolomics have been extensively applied. For instance, transcriptomic analysis of Litchi chinensis pericarp has provided insights into the molecular mechanisms underlying physiological changes during fruit ripening (Guo et al., 2018). Similarly, RNA-Seq technology has been utilized to investigate the transcriptional regulation of light-dependent anthocyanin accumulation in cherry fruits, comparing samples grown under illuminated and dark conditions (Tohge et al., 2005).

In maize, comparative transcriptome analyses of anthocyanin-pigmented kernels have identified WRKY transcription factors among the TFs showing increased expression in pigmented pericarp, suggesting potential involvement in anthocyanin regulation (Li et al., 2019). Metabolomic profiling of Arabidopsis overexpressing the MYB transcription factor gene PAP1 revealed specific accumulation of anthocyanins and quercetin derivatives. Among over 1,800 predicted metabolites in the PAP1-overexpressing lines, eight novel anthocyanins were identified (CHEN et al., 2022). Although metabolome–transcriptome studies related to anthocyanin accumulation have been reported in maize, comparative analyses across multiple fresh-eating maize cultivars with contrasting kernel colors remain limited. Here, we integrated metabolomic and transcriptomic data from four sweet maize cultivars to identify anthocyanin-associated metabolites, structural genes, and candidate transcription factors related to pigment accumulation. Importantly, this dataset provides a comparative framework for distinguishing shared and cultivar-associated molecular patterns, thereby extending current maize anthocyanin research beyond single-material observations. It also provides candidate targets and a useful resource for future functional validation and anthocyanin-oriented breeding.

## 2 Materials and methods

### 2.1 Plant materials

Four fresh-eating maize cultivars with contrasting kernel colors (WN 2000, HN, CN1, and HTN188) were grown at the Experimental Farm of Zhangjiakou Academy of Agricultural Sciences (Zhangjiakou, Hebei, China) under uniform agronomic management. The field trial was arranged as a randomized complete block design with three blocks (biological replicates), with each cultivar appearing once per block. Each plot consisted of four rows (row spacing 60 cm; plant spacing 30 cm) and was surrounded by guard rows. To minimize unintended pollination, ears were bagged before silking. These four cultivars exhibited distinct kernel color phenotypes, including white (WN 2000), yellow (HN), multicolored (CN1), and black (HTN188). From a pigment perspective, the differences among these accessions likely reflect variation not only in anthocyanin accumulation but also in carotenoid background. WN2000 showed no visible kernel pigmentation, whereas HN displayed a yellow phenotype consistent with carotenoid accumulation. CN1 exhibited mixed kernel coloration, while HTN188 showed strong black pigmentation indicative of substantial anthocyanin accumulation. However, the underlying Y1/y1 status (or equivalent carotenoid-related genotype) of these accessions, particularly HTN188, was not directly determined in this study.

Kernels were harvested at 15, 21, 27, and 33 days after silking (DAS). For each cultivar within each block, kernels were collected from approximately five representative plants/ears and pooled to form one biological replicate. For RNA-seq analysis, each pooled sample was treated as one biological replicate, and three biological replicates were included for each cultivar. No separate technical replicates were performed for RNA sequencing. This pooling strategy was used to reduce variation among individual plants within each block and to obtain a representative biological sample for each cultivar. Samples were immediately frozen in liquid nitrogen and stored at −80 °C until further analyses. For metabolomic and transcriptomic analyses, kernels collected at 15 DAS were used (4 cultivars × 3 biological replicates = 12 samples), as this stage represents a metabolically active phase of early kernel development and was considered suitable for capturing the initial molecular events associated with anthocyanin biosynthesis. In contrast, total anthocyanin content was monitored across all four developmental stages.

### 2.2 Total anthocyanin content

Samples stored at −80 °C were ground into fine powder in liquid nitrogen and extracted with 1 mL of a solvent mixture composed of methanol, ultrapure water, formic acid, and trifluoroacetic acid in a volume ratio of 70:27:2:1. After vortex mixing, samples were incubated at 4 °C for 24 h. The extract was centrifuged, and the supernatant was filtered for analysis. Absorbance was measured at 535 nm using an ultraviolet–visible spectrophotometer (Hong et al., 2020). A standard curve was generated using the equation: y = 0.0969x + 0.0288, where x represents the anthocyanin content and y denotes absorbance. Cyanidin chloride (Solarbio, China) was used as the reference standard. Total anthocyanin content was calculated from the calibration curve and expressed as cyanidin-3-glucoside (C3G) equivalents. Results are reported as mg C3G equivalents per kg dry weight (mg C3G eq/kg DW).

### 2.3 LC-MS/MS-based profiling of anthocyanin-related metabolites

Anthocyanin-related metabolites were analyzed using an ExionLC™ AD ultra-performance liquid chromatography system coupled with a QTRAP 6500+ tandem mass spectrometer (SCIEX, Framingham, MA, USA). Chromatographic separation was performed on an ACQUITY BEH C18 column (1.7 μm, 2.1 mm × 100 mm). The mobile phases consisted of ultrapure water containing 0.5% formic acid (solvent A) and methanol containing 0.5% formic acid (solvent B). The gradient elution program was as follows: 0.00 min, 5% B; 6.00 min, 50% B; 12.00 min, 95% B, held for 2 min; then returned to 5% B at 14.00 min and equilibrated for 2 min. The flow rate was 0.35 mL min^-1^, the column temperature was maintained at 40 °C, and the injection volume was 2 μL. Mass spectrometric detection was performed using an electrospray ionization (ESI) source in positive ion mode. The ion source temperature was set at 550 °C, the ion spray voltage was 5500 V, and the curtain gas was maintained at 35 psi. Data were acquired in multiple reaction monitoring (MRM) mode, and each ion pair was monitored using optimized declustering potential (DP) and collision energy (CE) parameters. This LC–MS/MS-based strategy has been widely used for anthocyanin characterization and quantification (Maldini et al., 2016; Steimer and Sjöberg, 2011). The full set of ion chromatograms is provided in Supplementary File S1 (ZIP archive).

### 2.4 RNA extraction, quantification, and sequencing

Total RNA from maize kernels was extracted using TRIzol reagent following the manufacturer’s protocol. A suitable amount of ground tissue was treated with 1 mL pre-cooled TRIzol and lysed at room temperature. Subsequently, chloroform and isopropanol were added for phase separation and RNA precipitation. The resulting RNA pellet was washed with 75% ethanol and dissolved in DEPC-treated water. RNA concentration and purity were assessed using a NanoDrop 2000 spectrophotometer, while RNA integrity was evaluated using the Agilent 5300 Bioanalyzer. Only high-quality RNA samples with OD260/280 values between 1.8 and 2.2 and RQN or RIN scores above 6.5 were used for library construction. For regular-input samples, libraries were prepared using the Illumina® Stranded mRNA Prep, Ligation kit; for low-input samples, the SMART-Seq_V4 Ultra Low Input RNA Kit was employed. The library construction process included poly(A) mRNA enrichment, RNA fragmentation, cDNA synthesis, end repair, adaptor ligation, PCR amplification, and purification. After quantification using Qubit 4.0, sequencing libraries were subjected to paired-end sequencing (PE150) on the Illumina NovaSeq X Plus platform (Kanehisa, 2019). The raw RNA-seq data generated in this study have been deposited in the NCBI Sequence Read Archive (SRA) under accession number PRJNA1300783.

### 2.5 Transcriptomic data analysis

Raw sequencing data were first processed using fastp to remove low-quality reads and adaptor contamination, generating high-quality clean reads (Chen et al., 2018). The filtered reads were aligned to the maize reference genome using HISAT2. Transcript assembly and expression estimation were performed with StringTie, and expression levels were summarized as TPM for downstream visualization (e.g., heatmaps) and descriptive analyses (Pertea et al., 2015). For differential expression analysis, raw read counts were obtained through the counting workflow implemented on the Majorbio Cloud Platform. Low-abundance transcripts were removed prior to DEG analysis by retaining only transcripts with counts ≥10 in at least 3 samples. Differentially expressed transcripts were then identified using DESeq2 based on the filtered raw count matrix (FDR <0.05 and |log_2_FC| ≥ 1) for all comparisons (three biological replicates per group). PCA was performed using DESeq2 variance-stabilized normalized counts to evaluate the similarity among biological replicates and the overall separation among cultivars. Gene Ontology (GO) annotation and KEGG pathway enrichment of differentially expressed transcripts were conducted using the Goatools package and Python’s scipy. stats module, respectively, with a significance threshold of P < 0.05. Some analyses were performed on the Majorbio Cloud Platform (cloud.majorbio.com) (Love et al., 2014; Shi et al., 2020; Huynh et al., 2019).

### 2.6 Weighted gene Co-expression network analysis (WGCNA)

Differentially expressed genes (DEGs) were used to construct the co-expression network by weighted gene co-expression network analysis (WGCNA), which was implemented on the Majorbio Cloud Platform using the platform-generated RSEM-FPKM expression matrix (genes filtered with FPKM ≥1 and coefficient of variation (CV) ≥ 0.1). A signed network was constructed with a soft-thresholding power of β = 9, minModuleSize = 30, minKMEtoStay = 0.3, and mergeCutHeight = 0.25. Module–trait relationships were evaluated using Spearman correlation. As external traits for module–trait correlation, we included the normalized abundance values of differentially accumulated metabolites (DAMs) derived from the LC–MS/MS dataset, particularly anthocyanin-related metabolites/features putatively annotated as malvidin-, petunidin-, peonidin-, cyanidin-, delphinidin-, and pelargonidin-related compounds, as well as the expression profiles of representative structural genes in the anthocyanin biosynthesis pathway (e.g., PAL, CHS, DFR, ANS, and UFGT). Key modules and candidate genes were prioritized based on module–trait correlations and intramodular connectivity.

### 2.7 Transcription factor prediction and filtering

Transcription factors (TFs) were predicted using the Majorbio Cloud Platform by querying PlantTFDB with the species set to Zea mays. TF annotation was performed using both sequence similarity search and domain-based identification with thresholds of BLAST E-value = 1e−5 and HMMER (hmmscan) E-value = 1e−5 (search against Zea mays, not all species). Differentially expressed TFs (DE-TFs) were defined as TF-annotated genes that were also identified as DEGs (FDR <0.05 and |log2FC| ≥ 1). Candidate TF regulators were prioritized by focusing on TF families known to participate in anthocyanin regulation (e.g., MYB and bHLH) and by network-based ranking (hub analysis) in the co-expression network analysis.

### 2.8 Transcription factor (TF) analysis

Transcription factors (TFs) were predicted on the Majorbio Cloud Platform by querying PlantTFDB with the species set to Zea mays, using thresholds of BLAST E-value = 1e−5 and HMMER (hmmscan) E-value = 1e−5. TFs were classified into families according to PlantTFDB annotations. To prioritize TF candidates potentially associated with anthocyanin biosynthesis, we focused on anthocyanin-associated TF families, including MYB, bHLH, and NAC, and constructed an interaction/co-expression network between candidate TFs and anthocyanin-pathway DEGs (n = 35). Hub candidates were identified based on the network topology ranking/centrality metrics implemented on the platform (Rice et al., 2000; Zhang et al., 2011).

### 2.9 Gene expression validation *via* quantitative real-time PCR (qRT-PCR)

To validate the reliability and accuracy of transcriptomic data, ten genes involved in anthocyanin biosynthesis were selected for qRT-PCR analysis. Expression levels were assessed in four maize varieties: WN 2000, HN, CN1, and HTN188. Total RNA was extracted using the TIANGEN Total RNA Extraction Kit (TIANGEN, Beijing, China), and cDNA synthesis was performed with the PrimeScript RT reagent kit with gDNA Eraser (TaKaRa, Kyoto, Japan). Specific primers for qRT-PCR were designed using Primer3 software (v2.5.0, https://primer3.ut.ee/). The qRT-PCR reactions were conducted using the TaKaRa SYBR Green Master Mix (TaKaRa, Beijing, China) on an ABI 7500 Fast Real-Time PCR System. Amplification was carried out in a 20 μL total reaction volume containing 10 μL 2× SYBR Premix Ex Taq, 0.4 μL ROX reference dye, 6 μL ddH_2_O, 0.8 μL forward primer, 0.8 μL reverse primer, and 2 μL cDNA template. The PCR program began with an initial denaturation at 95 °C for 30 s, followed by 40 cycles of amplification at 95 °C for 5 s, 60 °C for 35 s, 95 °C for 15 s, and 60 °C for 1 min. The relative expression levels were calculated using the 2^−ΔΔCT^ method, with internal reference genes serving as normalization controls (Hao et al., 2017).

### 2.10 Statistical analysis

For physiological/biochemical measurements (e.g., total anthocyanin content), data are presented as mean ± SD (n = 3). Differences among multiple groups were evaluated by one-way ANOVA followed by Duncan’s multiple range test (p < 0.05) using SPSS (v10.0). Differential metabolite analysis was performed in the metabolomics pipeline as described in Section 2.3 (VIP >1 and p < 0.05). Differential expression for RNA-seq was determined using DESeq2 based on raw read counts (FDR <0.05, |log2FC| ≥ 1), as described in Section 2.5.

## 3 Results and analysis

### 3.1 Phenotypic characteristics and total anthocyanin content of maize varieties WN 2000, HN, CN1, and HTN188

**FIGURE 1:**
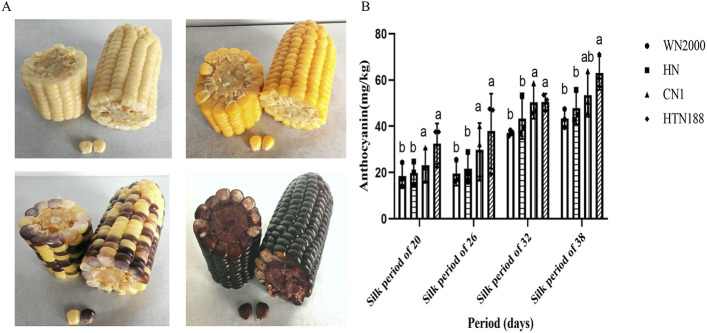
Anthocyanin Content in Maize Varieties of Different Colors. **(A)** Four fresh-eating maize varieties with different kernel colors and maturity stages were selected: white (WN 2000), yellow (HN), multicolored (CN1), and black (HTN188). **(B)** Anthocyanin content across developmental stages in the different colored maize varieties. According to Duncan’s multiple range test, bars labeled with different letters differ significantly at p < 0.05.

Metabolomic profiling was conducted on 12 samples, comprising three biological replicates from four maize cultivars of different kernel colors: white (WN 2000), yellow (HN), multicolored (CN1), and black (HTN188) (Figure 1A). Total anthocyanin content was measured across all four cultivars (Figure 1B), with values ranging from 32.487 to 63.026 mg/kg. Colored sweet maize exhibited significantly higher anthocyanin levels than the white and yellow varieties, and the black cultivar HTN188 showed the highest anthocyanin accumulation. As kernel development progressed, total anthocyanin content increased markedly in the pigmented cultivars (CN1 and HTN188), whereas the white (WN 2000) and yellow (HN) kernels maintained relatively low levels with only minor changes.

### 3.2 Global expression pattern analysis of samples

**FIGURE 2:**
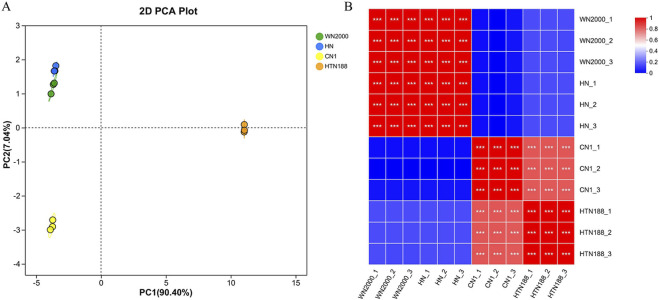
Comparative Analysis of Maize Samples with Different Kernel Colors. **(A)** PCA plot based on metabolomic data from 12 samples. PC1 and PC2 represent the first and second principal components, respectively; percentages indicate the variance explained by each component. Each point denotes an individual sample, and samples from the same group are colored identically. **(B)** Sample correlation heatmap based on metabolomic data from 12 samples.

A total of 12 samples from four maize cultivars, namely, white (WN 2000), yellow (HN), multicolored (CN1), and black (HTN188), were included in the metabolomic study, with three biological replicates for each cultivar. The principal component analysis (PCA) plot (Figure 2A) showed tight clustering of biological replicates within each cultivar, indicating good reproducibility. Notably, sample separation was dominated by PC1 (90.40%), which clearly distinguished HTN188 from the other cultivars, suggesting that HTN188 possesses the most distinct metabolomic profile. In contrast, WN2000 and HN clustered closely together in the PCA space, indicating highly similar metabolite profiles and supporting their grouping as a “low-pigment/low-anthocyanin” cluster rather than two well-separated inter-groups. Meanwhile, CN1 was positioned on the same side of PC1 as WN2000/HN but was separated along PC2 (7.04%), suggesting additional metabolic differences associated with its multicolored phenotype. Consistently, the sample correlation heatmap (Figure 2B) revealed high within-cultivar correlations and a higher similarity between WN2000 and HN, whereas CN1 and HTN188 formed another closely related cluster, with relatively lower correlations between the two clusters.

### 3.3 Metabolite profiling among maize varieties of different kernel colors

**FIGURE 3:**
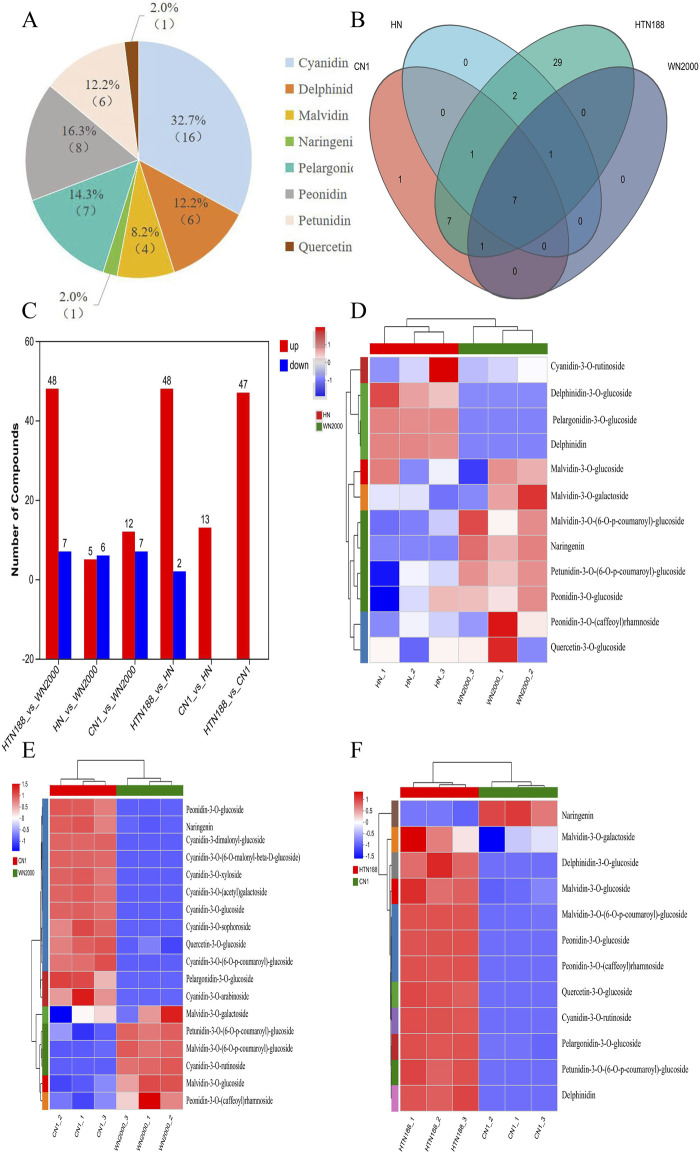
Metabolomic Analysis of Maize Cultivars with Different Kernel Colors. **(A)** Anthocyanin-related metabolites/features detected in the samples. **(B)** Venn diagram showing overlapping and unique differentially accumulated metabolites (DAMs) among different cultivars. **(C)** Comparison of DAMs across cultivar pairs. **(D,F)** Differentially accumulated anthocyanin-related metabolites in three pairwise comparisons: **(D)** HN vs. WN 2000, **(E)** CN1 vs. WN 2000, and **(F)** HTN188 vs. WN 2000. Cyanidin-related compounds were observed in CN1 and HTN188 but were not detected in WN2000 or HN.

LC–MS/MS-based metabolite profiling was performed to characterize anthocyanin-related metabolites/features in kernels of different colors. In total, 49 anthocyanin-related metabolites/features were detected and classified into different categories based on putative LC–MS/MS annotations (Figure 3A). The largest category was cyanidins (n = 16), followed by peonidins (n = 8), pelargonidins (n = 7), delphinidins (n = 6), petunidins (n = 6), malvidins (n = 4), naringenins (n = 1), and quercetins (n = 1). Venn diagrams (Figure 3B) were used to visualize the overlap of differentially accumulated metabolites (DAMs) among the cultivars. Seven DAMs were shared across all comparisons, while HTN188 exhibited 29 unique DAMs and CN1 showed one cultivar-specific DAM. Comparative analysis of six pairwise comparisons—HTN188 vs. WN 2000, HN vs. WN 2000, CN1 vs. WN 2000, HTN188 vs. HN, CN1 vs. HN, and HTN188 vs. CN1—revealed 48, 5, 12, 48, 13, and 47 upregulated DAMs, respectively (Figure 3C). These results suggest that HTN188 showed the most pronounced enrichment of anthocyanin-related metabolites among the four cultivars.

Pairwise comparisons against the white-kernel cultivar WN2000 further indicated cultivar-specific differences in anthocyanin-related metabolite accumulation. Specifically, the numbers of upregulated DAMs were five in HN vs. WN 2000, 12 in CN1 vs. WN 2000, and 48 in HTN188 vs. WN 2000 (Figure 3C), indicating that HTN188 showed the greatest increase in anthocyanin-related metabolites. Consistent with the DAM statistics, the differential metabolite profiles (Figures 3D–F) suggested that CN1 was mainly enriched in cyanidin- and peonidin-derived glycosides, such as cyanidin-3-O-glucoside and peonidin-3-O-glucoside, whereas HN displayed fewer upregulated DAMs and a relatively more noticeable increase in delphinidin-related compounds, such as delphinidin-3-O-glucoside. In contrast, HTN188 showed broader accumulation across multiple anthocyanin classes, including malvidins, delphinidins, and cyanidins, which was consistent with its black-kernel phenotype.

Among the putatively annotated metabolites, cyanidin-related compounds were observed in HTN188 and CN1, but not in WN2000 or HN. In addition, petunidin-related compounds were observed only in HTN188 (Supplementary Table 1), suggesting that these metabolites may be associated with kernel color variation among the maize cultivars. Overall, differences in the accumulation patterns of anthocyanin-related metabolites may contribute to the observed variation in kernel color.

### 3.4 Transcriptome sequencing, assembly, and statistics

To further investigate the mechanisms underlying kernel color formation in maize, RNA-seq–based transcriptome profiling was performed on four maize cultivars with contrasting kernel colors, with three biological replicates per cultivar. RNA-seq generated a total of 97.26 Gb of clean data, with individual libraries producing 40,278,334–51,044,728 raw reads. After quality filtering and adaptor trimming, 39,906,574–50,621,658 clean reads were retained per sample. The average GC content was 49.59%, and >95.27% of bases had Q30 scores (Supplementary Table 2). Clean reads were aligned to the maize reference genome using HISAT2, with an overall mapping rate of 87.72%–90.65%, including 81.46%–86.43% uniquely mapped reads and 3.26%–8.03% multi-mapped reads (Supplementary Table 3). PCA of the RNA-seq samples showed that biological replicates clustered closely within each cultivar, indicating good reproducibility (Supplementary Figure 1). Transcripts were reconstructed in a reference-guided manner using StringTie, yielding 179,447 transcripts with an average length of 1,472 bp; among them, 98,331 transcripts were ≥1,800 bp (Supplementary Table 4). In total, 46,430 transcripts were annotated using six public databases (NR, GO, KEGG, Swiss-Prot, Pfam, and EggNOG), and 92.17% had at least one annotation (Supplementary Table 5), with 91.13% matched in the NR database. Collectively, these results indicate that the RNA-seq data and genome alignments are of high quality and suitable for downstream analyses.

### 3.5 Transcriptomic analysis of maize varieties with different kernel colors

**FIGURE 4:**
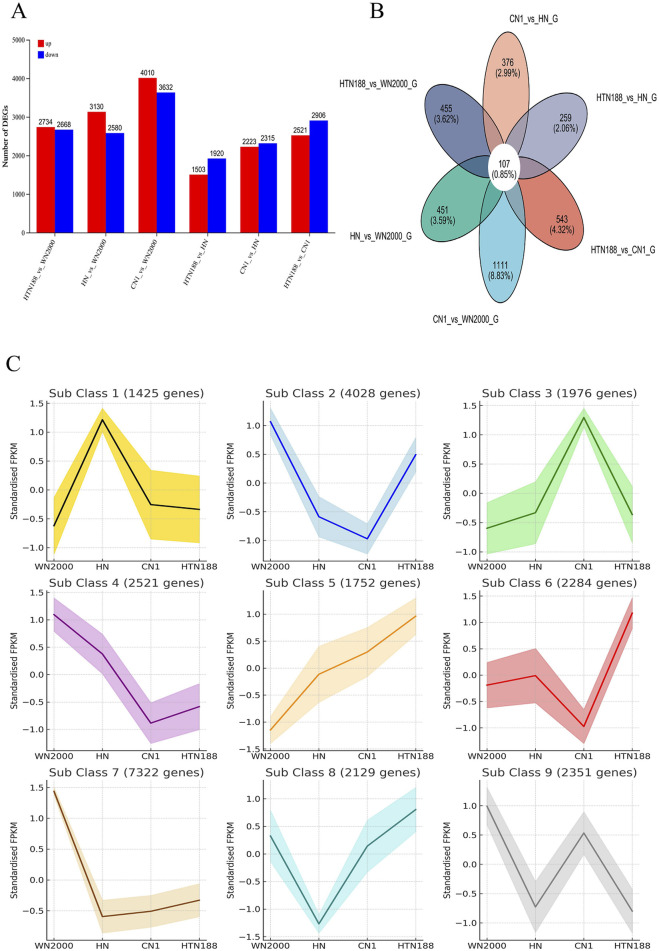
Transcriptomic Analysis of Four Maize Groups (Three Biological Replicates Each). **(A)** Comparison of DEGs among cultivars. **(B)** Venn diagram showing shared and unique DEGs among the six pairwise cultivar comparisons. **(C)** Expression patterns of DEGs. The x-axis represents samples; the y-axis shows normalized, centralized FPKM expression values.

To identify key differentially expressed genes (DEGs) involved in anthocyanin biosynthesis, six pairwise comparisons were conducted: HTN188 vs. WN 2000, HN vs. WN 2000, CN1 vs. WN 2000, HTN188 vs. HN, CN1 vs. HN, and HTN188 vs. CN1 (Figure 4). In total, 12,557 DEGs were identified across all samples. Among the comparisons, CN1 vs. WN2000 revealed the highest number of DEGs (7,642), with 4,010 genes upregulated and 3,632 downregulated (Figure 4A). Conversely, HTN188 vs. HN yielded the lowest number of DEGs (3,423), including 1,503 upregulated and 1,920 downregulated genes (Figure 4A). The relationships among DEGs in the six groups were visualized using a Venn diagram (Figure 4B), which revealed 107 commonly expressed genes and 3,195 distinct DEGs. These may play important roles in the regulation of anthocyanin biosynthesis and accumulation across different maize cultivars. To analyze gene expression trends among the different varieties, FPKM values were first centralized and normalized, followed by K-means clustering. Based on the K-means clustering results (Figure 4C), DEGs were grouped into nine distinct clusters (Supplementary Table 6). Cluster five showed a gradual increase in gene expression from white to darker-colored varieties, consistent with anthocyanin accumulation trends. These genes may serve as potential markers for distinguishing pigment-related phenotypes in maize.

### 3.6 Functional annotation of differentially expressed genes (DEGs)

**FIGURE 5:**
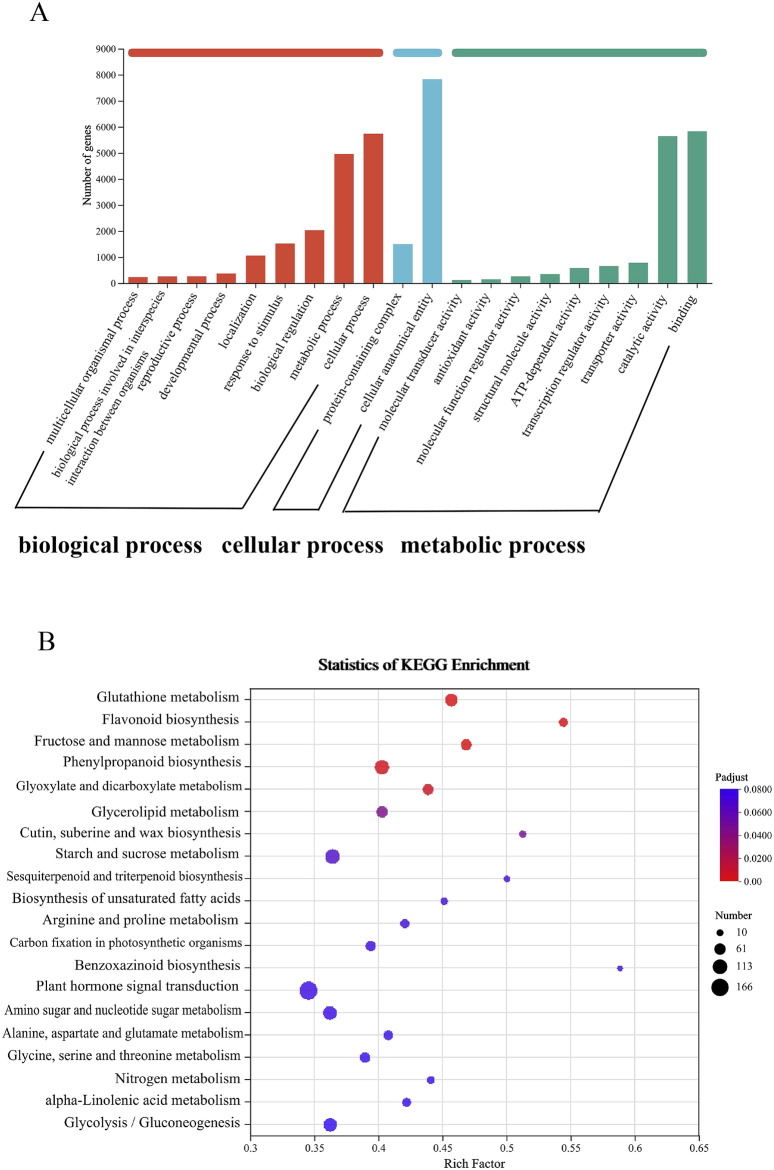
GO and KEGG Enrichment Analyses of DEGs. **(A)** GO enrichment analysis of all DEGs. **(B)** Top 20 KEGG pathways enriched based on p-value significance.

To clarify the functions of DEGs and identify genes involved in regulating anthocyanin accumulation in maize, all DEGs were first subjected to Gene Ontology (GO) analysis. In the biological process category, most DEGs were assigned to the terms “biological process” (5,734 genes, 34.64%), “metabolic process” (4,958 genes, 29.95%), and “cellular process” (2,028 genes, 12.25%). In the cellular component category, enrichment was observed in terms such as “protein-containing complex” and “cellular anatomical entity.” For molecular function, approximately 72.5% of DEGs were mapped to “catalytic activity” and “binding” (Figure 5A). KEGG enrichment results were presented as scatter plots, where enrichment levels were assessed based on the rich factor, Q-value, and the number of DEGs associated with each pathway (Figure 5B). The rich factor refers to the ratio of DEGs to the total number of genes annotated in a given pathway. A higher rich factor indicates greater enrichment, and a smaller Q-value denotes stronger statistical significance.

In total, 4,078 DEGs across all samples were mapped to 133 KEGG pathways (Supplementary Table 7). Comparative analysis among the four colored maize samples revealed significant enrichment of DEGs in pathways such as “phenylpropanoid biosynthesis,” “glutathione metabolism,” “fructose and mannose metabolism,” “glyoxylate and dicarboxylate metabolism,” and “flavonoid biosynthesis” (Figure 5B). Notably, the “fructose and mannose metabolism” and “glutathione metabolism” pathways were significantly enriched across all comparisons. The “flavonoid biosynthesis” pathway was enriched specifically in HTN188 vs. HN, HTN188 vs. CN1, and CN1 vs. HN. In contrast, the “plant hormone signal transduction” pathway was enriched in HN vs. WN2000 and CN1 vs. WN 2000 (Supplementary Figure 2). These results suggest that the “flavonoid biosynthesis” pathway appears to be more active in the darker-colored maize varieties, whereas the “plant hormone signal transduction” pathway is more prominently activated in the group associated with WN 2000.

### 3.7 Identification of key DEGs in the anthocyanin biosynthesis pathway

**FIGURE 6:**
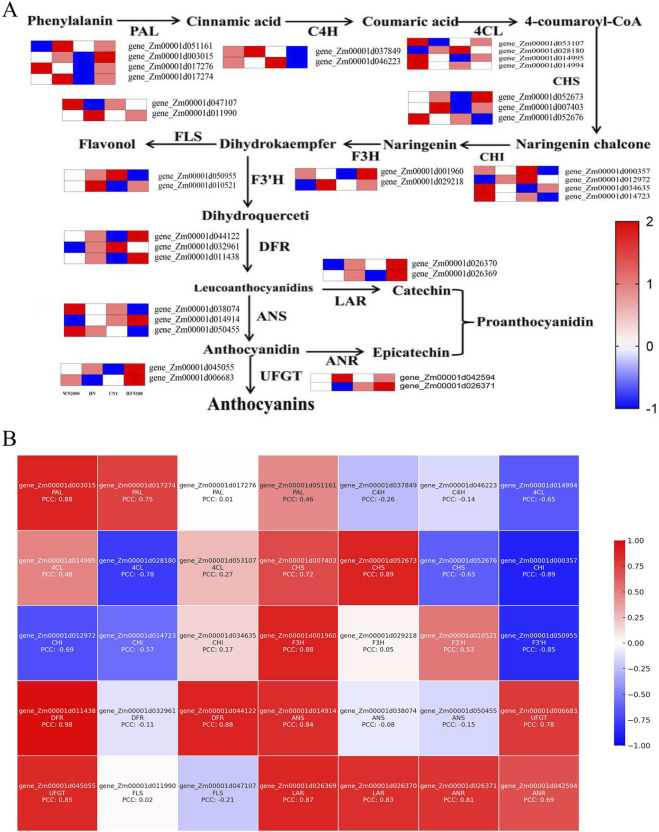
Analysis of DEGs in the Anthocyanin Biosynthesis Pathway in Maize. **(A)** Heatmap illustrating log_2_-transformed FPKM values of DEGs involved in the anthocyanin biosynthesis pathway and structural enzyme genes. Enzymes include 4-coumarate-CoA ligase (4CL), phenylalanine ammonia-lyase (PAL), trans-cinnamate 4-monooxygenase (C4H), chalcone synthase (CHS), flavanone 3-hydroxylase (F3H), chalcone isomerase (CHI), flavonoid 3′-hydroxylase (F3′H), dihydroflavonol 4-reductase (DFR), flavonol synthase (FLS), UDP-flavonoid glucosyltransferase (UFGT), anthocyanidin synthase (ANS), anthocyanidin reductase (ANR), and leucoanthocyanidin reductase (LAR). **(B)** Pearson correlation analysis between anthocyanin biosynthesis-related gene expression and total anthocyanin content. Positive and negative correlations are indicated by different colors. Samples: WN 2000 (white maize), HN (yellow maize), CN1 (multicolored maize), HTN188 (black maize). The heatmap color gradient reflects gene regulation: red for upregulation, white for no change, and blue for downregulation.

To elucidate the molecular basis underlying differences in anthocyanin biosynthesis among four maize cultivars with contrasting kernel colors, 35 DEGs were enriched in the anthocyanin biosynthetic pathway, including PAL, C4H, 4CL, CHS, CHI, F3H, F3′H, DFR, ANS, UFGT, FLS, ANR, and LAR (Figure 6A). Because these enzymes are encoded by multigene families in maize, we examined the specific gene members and observed clear cultivar-dependent patterns. For example, the black-kernel cultivar HTN188 showed markedly higher expression of several late biosynthetic gene members (e.g., ANS gene_*Zm00001d014914* and DFR gene_*Zm00001d044122*) than WN 2000, consistent with enhanced anthocyanin accumulation (Figure 6A; Supplementary Table 8). Pearson correlation analysis between pathway-gene expression and total anthocyanin content (Figure 6B) showed that 22 genes were positively correlated and 13 genes were negatively correlated (P < 0.05; |PCC| > 0.8). Notably, gene_*Zm00001d011438* exhibited a strong positive correlation with total anthocyanin content, suggesting it as a key candidate associated with anthocyanin accumulation.

### 3.8 Correlation analysis between WGCNA modules and major traits

**FIGURE 7:**
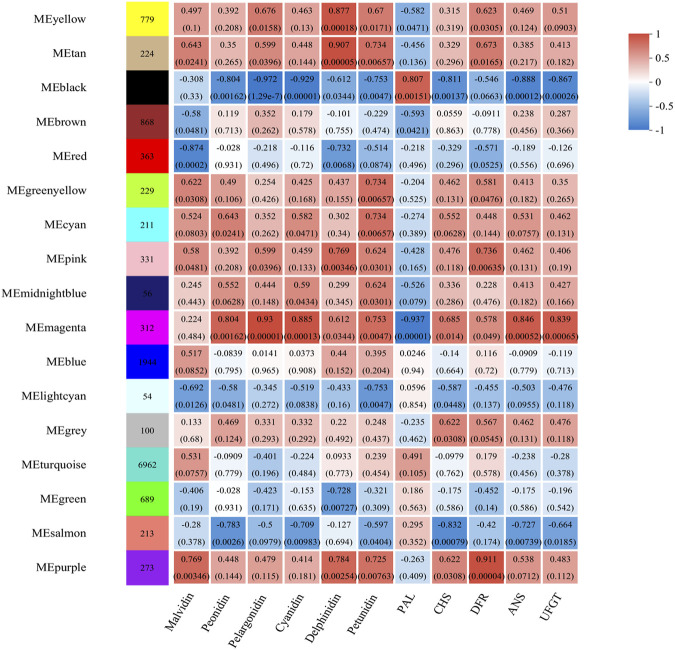
Correlation Analysis Between WGCNA Modules and Key Traits. The left panel displays 17 WGCNA modules. The right panel shows module–trait correlations with putatively annotated anthocyanin-related metabolite traits derived from the LC–MS/MS dataset, together with representative structural genes in the anthocyanin biosynthesis pathway. The color scale indicates module–trait correlations, ranging from −1 (blue) to 1 (red), with corresponding p-values.

As shown in Figure 7, a total of 17 expression modules were identified through WGCNA, including modules such as MEyellow, MEbrown, and MEturquoise. Spearman correlation coefficients were calculated between each module and LC–MS/MS-derived anthocyanin-related metabolite traits, particularly metabolites putatively annotated as malvidin-, peonidin-, and pelargonidin-related compounds, as well as the expression levels of representative structural genes in the anthocyanin biosynthesis pathway (e.g., DFR, ANS, and UFGT) (Figure 7; Supplementary Table 9). The results revealed diverse correlation patterns between modules and traits. For instance, the MEblack module showed a highly significant negative correlation with the pelargonidin-related trait (r = −0.972, p = 1.29e−7), suggesting that genes within this module may be negatively associated with pelargonidin accumulation. In contrast, the MEmagenta module was significantly positively correlated with the cyanidin-related trait (r = 0.885, p = 0.00013), ANS (r = 0.846, p = 0.00052), and UFGT (r = 0.839, p = 0.00065), suggesting that this module contains candidate genes associated with anthocyanin biosynthesis and regulation. Other modules, such as MEgreen and MEsalmon, displayed weak or non-significant correlations with most traits, suggesting a limited or indirect association with the traits analyzed in this study.

### 3.9 Prediction of transcription factor regulation of anthocyanin expression

**FIGURE 8:**
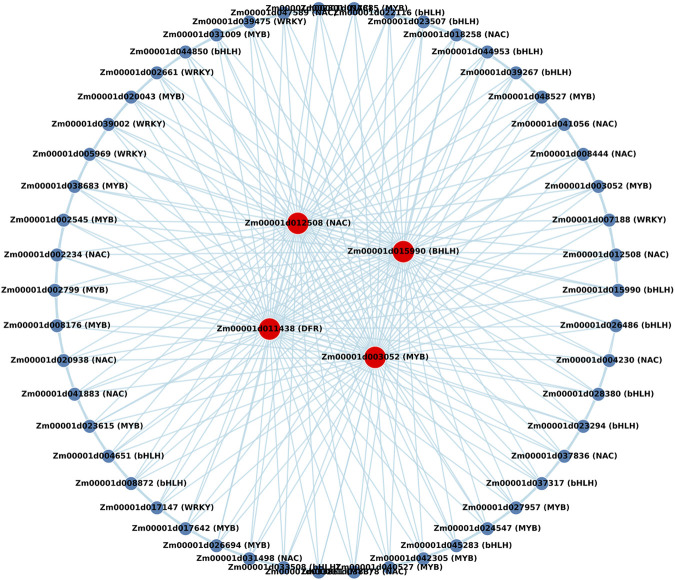
Interaction Network of Key Structural Enzyme Genes and Transcription Factors (TFs) in Maize Anthocyanin Biosynthesis. Red nodes represent hub genes; blue nodes denote co-expressed genes with relatively weaker correlations.

To investigate whether transcription factors (TFs) regulate anthocyanin biosynthesis and accumulation in different-colored maize cultivars, TF prediction analysis was conducted. In total, 6,928 TF-annotated transcripts (putative TF-encoding ORFs detected in our RNA-seq data) were identified by querying PlantTFDB (Zea mays) on the Majorbio Cloud Platform (Supplementary Table 10). Classification revealed that most TFs belonged to the MYB, C2C2, AP2/ERF, C2H2, and bHLH families. Previous studies have shown that bHLH, MYB, and WD40 TFs play key roles in regulating anthocyanin biosynthesis by activating or repressing the transcription of structural genes. Therefore, we performed interaction network analysis of candidate TFs, including MYB, bHLH, and NAC family members, with 35 DEGs involved in the anthocyanin biosynthesis pathway (Figure 8), aiming to identify hub TF genes that may influence anthocyanin biosynthesis. The analysis identified one hub structural gene, DFR (*Zm00001d011438*), and three candidate hub transcription factors, including MYB (*Zm00001d003052*), bHLH (*Zm00001d015990*), and NAC (*Zm00001d012508*), that were associated with anthocyanin accumulation (Supplementary Table 11).

### 3.10 Integrated analysis of metabolomic and transcriptomic data

**FIGURE 9:**
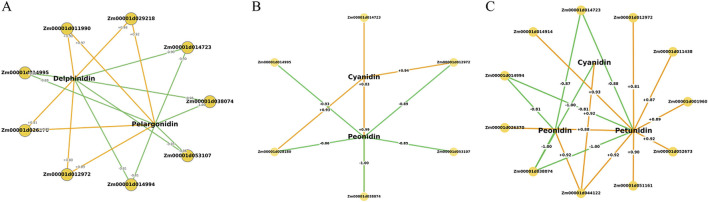
Correlation Between DEGs and Differential Metabolites Related to Anthocyanin Biosynthesis. **(A)** HN vs. WN 2000. **(B)** CN1 vs. WN 2000. **(C)** HTN188 vs. WN2000. Correlation lines indicate the degree of association between differentially expressed genes and anthocyanin metabolites, with numerical labels representing correlation coefficients.

Using WN2000 as a reference control, the transcript-metabolite correlation analysis was performed across three comparisons. In the HN vs. WN2000 group, nine genes—including one ANS, two CHI, one F3H, one FLS, three 4CL, and one LAR-were found to be associated with pelargonidin and delphinidin accumulation (Figure 9A). Among them, four genes were positively correlated with delphinidin, while five genes were negatively correlated with pelargonidin. In the CN1 vs. WN2000 group, six genes—including one ANS, three 4CL, one CHI, and one CHS—were associated with cyanidin and peonidin metabolism (Figure 9B). Notably, *Zm00001d028180* and *Zm00001d012972* were positively correlated with cyanidin but negatively correlated with peonidin. In the HTN188 vs. WN2000 comparison, 11 genes—including two ANS, two DFR, two CHI, one 4CL, one PAL, one CHS, one F3H, and one LAR-were correlated with cyanidin, peonidin, and petunidin biosynthesis (Figure 9C). Among them, *Zm00001d044122* showed positive correlations with all three metabolites, whereas *Zm00001d038074* showed negative correlations across the board. Notably, ANS, CHI, and 4CL genes were present in all three comparisons, suggesting their critical involvement in anthocyanin accumulation.

### 3.11 Validation of RNA-Seq data *via* qRT-PCR

To validate the accuracy of the RNA-Seq data, ten genes were selected for qRT-PCR to assess their expression levels in WN 2000, HN, CN1, and HTN188 (Table 1). The relative expression trends of these candidate genes were consistent with RNA-Seq results, indicating high reliability and accuracy of the transcriptomic data. Further linear regression analysis revealed strong correlations between gene expression levels obtained by qRT-PCR and those from RNA-Seq (Supplementary Figure 3, *R* ^2^ > 0.82), confirming the credibility of the RNA-Seq dataset.

**TABLE 1:** qRT-PCR Primer sequence.

Gene ID	Forward primer (5′–3′)	Reverse primer (5′–3′)
gene_*Zm00001d017274*	TGC​TCT​TCT​TGG​AGG​GAA​ACG	AAC​GTG​CTG​GAC​TCA​CCT​AA
gene_*Zm00001d028180*	TGG​TCT​CAA​CGA​CAC​GAT​GG	CCC​TCC​ATG​ACA​CAG​GCA​AT
gene_*Zm00001d014995*	AGT​CCA​TCA​TCG​TCG​CAG​TC	TCG​TTC​TCG​ACC​TCT​CGT​CT
gene_*Zm00001d034635*	GCC​AAA​GAA​GGG​TCA​GGT​CA	TTT​GAG​GGT​CCA​TTC​CCG​TG
gene_*Zm00001d044122*	CGA​GAC​AAA​CTC​ACC​GGG​AT	GAA​AGG​GAA​GGT​GCT​GGT​CA
gene_*Zm00001d032961*	ATT​CAA​CGG​GGG​AGG​TAA​GC	TGG​GGA​CTG​AAA​CGG​TGA​TG
gene_*Zm00001d010521*	ATT​TAG​TGG​GCC​TCG​GTG​TG	AAC​CTA​ATT​GTG​GGC​CTG​GG
gene_*Zm00001d047107*	GGG​CGA​TGA​GCA​ACA​CAT​TC	GGA​CAT​CCC​ACA​ATG​GAG​CA
gene_*Zm00001d050455*	TAG​TAG​TAG​ACC​AGC​GGC​GT	ATG​ACG​AAG​CAA​GTG​GTG​GT
gene_*Zm00001d026370*	AAA​GTC​CAG​TCC​TGA​TCG​CC	GGT​ACA​GGA​CGC​TGG​ATG​AC
Gapdh	GGT​GGT​GCT​AAG​CGT​GTG​AA	GCC​AAA​TTC​GTT​GTC​GTA​CC

## 4 Discussion

### 4.1 Variations in anthocyanin content among maize varieties of different colors

Anthocyanins are water-soluble phenolic pigments responsible for red, purple, blue, and even black coloration in fruits, vegetables, cereals, flowers, and other pigmented plant tissues (Knievel et al., 2009; Rodriguez-Amaya, , 2019). Numerous studies have reported correlations between seed coat color and agronomic or nutritional traits, including seed vigor, grain nutritional quality, and antioxidant capacity (Tsuda et al., 1994; Li et al., 2014; Malik et al., 2003; Singletary et al., 2003; Nakaishi et al., 2001; Canter and Ernst, 2004). Anthocyanins are typically stored in vacuoles, and their accumulation patterns can vary with tissue type and developmental stage (Jansen and Flamme, 2006; Boss et al., 1996). In sweet maize, total anthocyanin content generally increases during kernel development, with rapid increases at early stages followed by a slower rise at later stages (Lewis et al., 2016; Frangne et al., 2002; Kim et al., 2020). In the present study, total anthocyanin content was quantified across developmental stages in four sweet maize cultivars with contrasting kernel colors. The light-colored cultivars (WN2000 and HN) showed similarly low anthocyanin levels, whereas the pigmented cultivars (CN1 and especially HTN188) exhibited markedly higher anthocyanin accumulation. These results indicate that anthocyanin accumulation is closely associated with pigmentation in CN1 and HTN188. However, the clear visual difference between WN2000 and HN, despite the lack of a significant difference in total anthocyanin content, suggests that pigments other than anthocyanins, most likely carotenoids, are major contributors to the color difference between these two cultivars. Therefore, the present study should be interpreted as focusing primarily on anthocyanin-associated metabolic and transcriptional differences rather than as a complete explanation of all kernel color variation across the four accessions.

### 4.2 Identification of anthocyanin metabolites

Anthocyanins widely distributed in nature are mainly derived from three core anthocyanidins: pelargonidin, cyanidin, and delphinidin. From these, three additional derivatives are formed *via* methylation: peonidin from cyanidin, petunidin from delphinidin, and malvidin from further methylation of petunidin. Purple maize from Peru contains primarily cyanidin-3-O-β-D-glucoside, pelargonidin-3-O-β-D-glucoside, peonidin-3-O-β-D-glucoside, cyanidin-3-O-β-D-(6-malonyl-glucoside), pelargonidin-3-O-β-D-(6-malonyl-glucoside), and peonidin-3-O-β-D-(6-malonyl-glucoside). Similarly, Andean purple maize contains pelargonidin-3-glucoside and peonidin-3-glucoside, along with their respective malonylated derivatives (de Pascual et al., 2017). In black rice, major anthocyanidins identified include cyanidin-3-glucoside, peonidin-3-glucoside, cyanidin-3,5-diglucoside, and cyanidin-3-rutinoside (Hou et al., 2013). Among 15 Mexican blue maize varieties, two main types of anthocyanidins were found: cyanidins and pelargonidins. Acylated anthocyanidins were especially abundant in blue maize, including cyanidin-3-(6′-disuccinyl-glucoside) (Cy-diSuc-Glu), cyanidin-3-(6′-succinyl-glucoside) (Cy-Suc-Glu), cyanidin-3-glucoside (Cy-3-Glu), and pelargonidin-3-glucoside (Py-3-Glu) (Mora-Rochín et al., 2016). In purple supersweet maize, 20 anthocyanidins were identified, mainly cyanidins, peonidins, and pelargonidins, with cyanidins accounting for 77% of total anthocyanins—significantly higher than the other two types (Hong et al., 2010). Analysis of U.S. blue maize varieties revealed significant differences in anthocyanin composition among cultivars and growing regions. Cyanidin-3-glucoside was the predominant compound, followed by pelargonidins and peonidins, and content variation among genotypes ranged from 1- to 6-fold (Nankar et al., 2016). Our study confirmed that HTN188 contained the most abundant and diverse anthocyanin compounds, with significantly more upregulated metabolites than other varieties (Figure 3). Our metabolomics results showed that malvidins were present in all four cultivars and were dominant in WN2000 and HN. Cyanidins were enriched mainly in CN1 and HTN188, while HTN188 exhibited the highest levels of cyanidin, peonidin, and pelargonidin. These three classes are likely responsible for the black kernel phenotype, consistent with Chatham’s findings (Chatham et al., 2018).

### 4.3 Molecular characterization of key genes associated with anthocyanin accumulation in sweet maize

In plants, phenylpropanoids represent an important group of physiologically active secondary metabolites derived from phenylalanine. Anthocyanins, flavonols, isoflavones, and flavones share similar biosynthetic pathways during their formation (Chaves-Silva et al., 2018). The results of this study revealed that, in comparisons among four colored maize samples, darker-colored maize relied more heavily on the phenylalanine pathway and its branches—the flavonoid and flavonol biosynthesis pathways. The phenylalanine pathway is one of the core routes of plant secondary metabolism, producing a series of phenolic acids and flavonoid antioxidants that play critical roles in plant stress resistance. In contrast, the glutathione biosynthesis pathway was more active in light-colored maize, a route primarily involved in scavenging free radicals and regulating immunity. Overall, the color differences were accompanied by selective activation of distinct branches of the secondary metabolic network: light-colored maize tended to favor terpenoid/glutathione pathways, while dark-colored maize preferentially activated phenylpropanoid/flavonoid pathways. Such differences in functional pathways not only uncover the biochemical mechanisms underlying the accumulation of functional components in maize of different colors but also reflect, from an evolutionary perspective, the trade-offs in resource allocation—plants balance growth and defense by regulating pathway activity, thereby leading to nutritional and functional differentiation among varieties.

In the anthocyanin biosynthesis pathway, DFR catalyzes the conversion of dihydroflavonols to leucoanthocyanidins (Anirban et al., 2023). Previous studies have shown that the introduction of maize DFR into white-flowered Petunia hybrida resulted in a novel brick-red flower phenotype, while the transcript levels of TaDFR in purple wheat were closely associated with anthocyanin content (Banerjee et al., 2023; Grotewold, 2006). In the present study, key structural genes involved in anthocyanin biosynthesis, including PAL, CHS, F3H, DFR, and UFGT, were upregulated in HTN188. Accordingly, the increased anthocyanin content in black sweet maize is likely associated with the elevated expression of these genes. Notably, one DFR gene (*Zm00001d011438*) was highly correlated with total anthocyanin content (|PCC| > 0.8). As a structural gene encoding a key enzyme in the anthocyanin biosynthesis pathway rather than a transcription factor, its identification as a hub gene likely reflects its central metabolic position and strong co-expression with regulatory genes. Phylogenetic analysis revealed that the amino acid sequence of maize DFR exhibited high homology with those of barley (78.26%), rice (76.92%), and wheat (79.13%) DFR proteins (Supplementary Table 12), indicating a highly conserved function. Previous studies have shown that DFR genes in these species catalyze the reduction of dihydroflavonols to leucoanthocyanidins, thereby influencing anthocyanin accumulation and tissue pigmentation (Ferrer et al., 2008; Nakatsuka et al., 2003). Therefore, *Zm00001d011438* is a strong candidate structural gene associated with anthocyanin accumulation and pigmentation differences, particularly in the anthocyanin-rich cultivars. However, although DFR represents a key biosynthetic gene closely associated with anthocyanin accumulation, the differentially expressed transcription factors identified in the co-expression network are more likely to function as upstream regulators underlying the observed variation in anthocyanin accumulation among cultivars. Furthermore, both positively and negatively correlated modules with anthocyanin content were identified in the co-expression network. One hub structural gene *(Zm00001d011438*) and three candidate hub transcription factors, namely, MYB (*Zm00001d003052*), bHLH (*Zm00001d015990*), and NAC (*Zm00001d012508*), were highlighted, and their expression patterns paralleled the trends in total anthocyanin content. Conversely, transcription factors showing negative correlations with anthocyanin content may represent candidate negative regulators or upstream-associated components involved in reduced anthocyanin accumulation, although this interpretation requires further functional validation. Mechanistically, anthocyanin production is widely controlled at the transcriptional level by TF modules such as the MYB–bHLH–WD40 (MBW) complex. Our TF/network analyses prioritized MYB, bHLH and NAC candidates associated with anthocyanin-rich phenotypes, and correlation/WGCNA further highlighted candidate regulators and pathway genes whose expression tracked anthocyanin accumulation. Importantly, these results provide a data-driven shortlist of candidate regulators and biosynthetic genes for future functional validation rather than definitive proof of causality.

### 4.4 Limitations and future perspectives

We acknowledge that a limitation of this study is that metabolomic and transcriptomic analyses were performed only at 15 DAS, whereas total anthocyanin content was monitored across four developmental stages. We selected 15 DAS because it represents a metabolically active stage of early kernel development and is suitable for capturing early molecular events associated with anthocyanin biosynthesis. Thus, our omics data mainly reflect the initiation or early-stage accumulation of anthocyanins, and regulators acting predominantly at later developmental stages may not have been captured. In addition, the WGCNA analysis was performed using the platform-generated RSEM-FPKM expression matrix. Although count-based transformed data are often considered more suitable for correlation network analysis, the identified co-expression modules and hub genes in this study should be interpreted as candidate associations that warrant further validation. We also acknowledge that our conclusions regarding regulators are primarily based on expression correlation and network prioritization and therefore do not establish direct causality. Future work should validate TF–target relationships and key structural genes (e.g., *Zm00001d011438*) using functional assays such as transient activation, genetic perturbation, and promoter-binding analyses (e.g., Y1H/ChIP-qPCR). Targeted LC–MS quantification of major anthocyanins across developmental stages would further refine the links between gene expression and anthocyanin composition.

## 5 Conclusion

In summary, the black maize variety HTN188 exhibited markedly higher anthocyanin content than the other cultivars. Integrated transcriptomic and metabolomic analyses of four fresh-eating maize cultivars with contrasting kernel colors revealed that anthocyanin-associated differences were especially evident in the pigmented cultivars CN1 and HTN188, whereas the visual difference between WN2000 and HN is likely influenced by pigments other than anthocyanins. The black kernel phenotype of HTN188 was associated with increased accumulation of cyanidin-, peonidin-, and pelargonidin-related compounds. Among the differentially expressed structural genes involved in the anthocyanin biosynthesis pathway, higher expression of F3H, DFR, and UFGT was closely associated with anthocyanin accumulation in HTN188. In particular, the expression level of *Zm00001d011438* showed a significant positive correlation with total anthocyanin content, suggesting that this gene may represent an important candidate structural gene associated with anthocyanin accumulation. WGCNA further identified co-expression modules associated with anthocyanin accumulation, as well as candidate transcription factors, including MYB, bHLH, and NAC, that may contribute to anthocyanin-related regulatory variation. Overall, this study provides a comparative multi-omics perspective on anthocyanin accumulation in fresh-eating maize and highlights candidate structural and regulatory genes for future functional validation and genetic improvement.

## Data Availability

The original contributions presented in the study are publicly available. This data can be found in the NCBI SRA repository with the accession number PRJNA1300783.

## Supplementary material

SUPPLEMENTARY FIGURE S1 PCA score plot of samples from four maize cultivars (CN1, HN, HTN188, and WN2000) based on LC–MS features of anthocyanins/anthocyanin-related metabolites. Each point represents one biological replicate (n = 3). PC1 and PC2 explain 52.77% and 14.64% of the total variance, respectively.

SUPPLEMENTARY FIGURE S2 KEGG pathway enrichment of differential metabolites among four maize cultivars. Panels: **(A)** HTN188 vs. WN 2000; **(B)** HTN188 vs. HN; **(C)** HTN188 vs. CN1; **(D)** CN1 vs. WN 2000; **(E)** CN1 vs. HN; **(F)** HN vs. WN 2000. The x-axis indicates the rich factor and the y-axis lists KEGG pathways. Bubble size represents the number of differential metabolites mapped to each pathway, and bubble color represents the adjusted p value.

SUPPLEMENTARY FIGURE S3 Validation of RNA-seq results by qRT-PCR. Left: comparison of expression changes for seven representative genes (Gene IDs shown), with RNA-seq log₂(FPKM) (log₂FC) in black and qRT-PCR log₂(FC) in gray. Right: correlation between qRT-PCR and RNA-seq data with linear regression (dotted line); the regression equation and R² (0.8275) are shown.
