# Supplementary figures and images for: Integrated metabolomic and transcriptomic analysis of anthocyanin accumulation mechanisms in maize kernels of different colors
Source: Front Genet. 2026 Jun 1;17:1797093. doi: 10.3389/fgene.2026.1797093 (PMC13265062; doi:10.3389/fgene.2026.1797093)

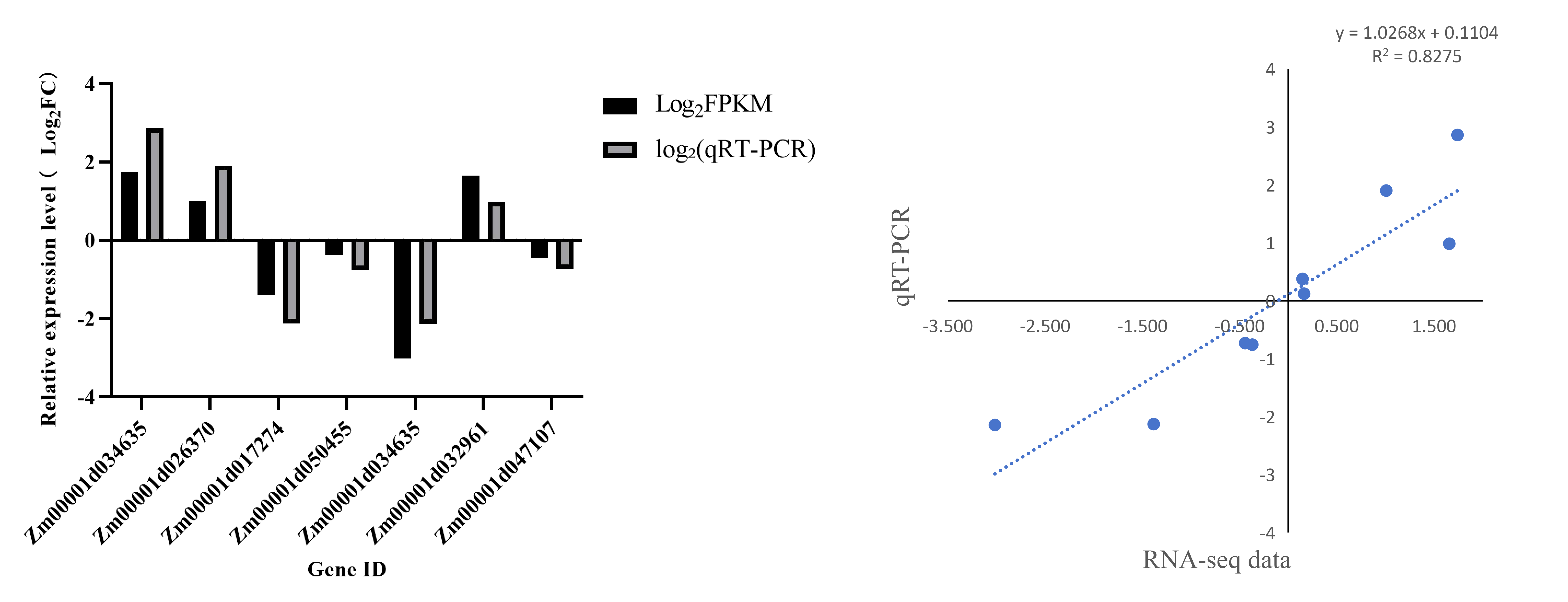

Supplement: Supplementary file 1 [file DataSheet1.zip › Supplement/Supplementary Figure 3.tif]

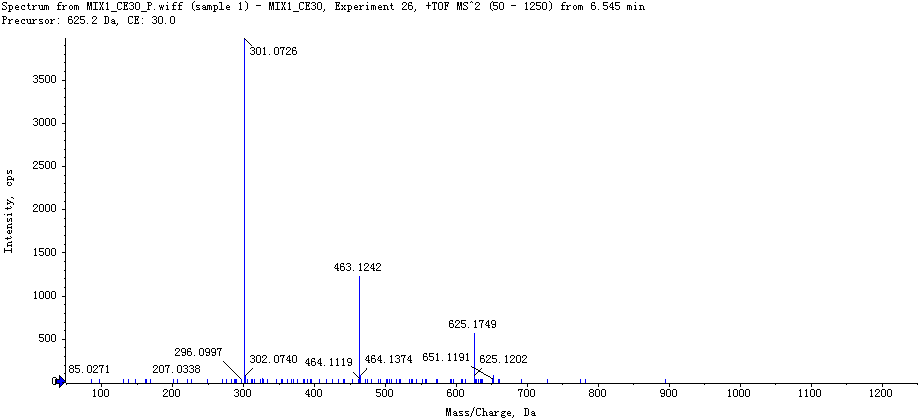

Supplement: Supplementary file 2 [file DataSheet2.zip › Peonidin-3,5-O-diglucoside.png]

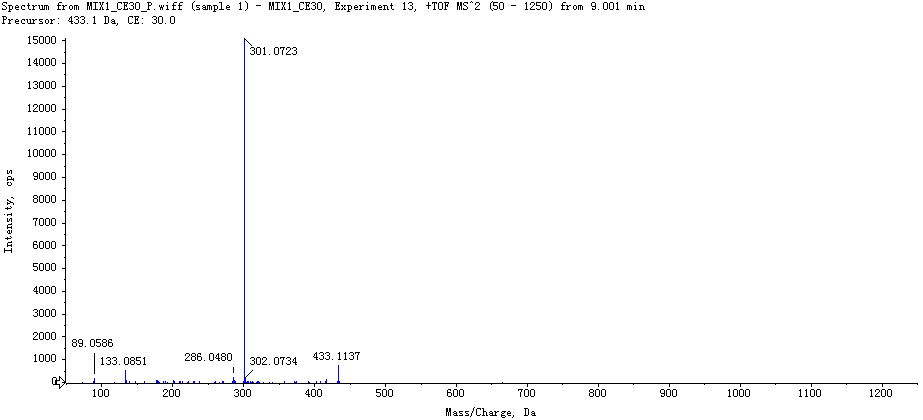

Supplement: Supplementary file 2 [file DataSheet2.zip › Peonidin-3-O-arabinoside.png]

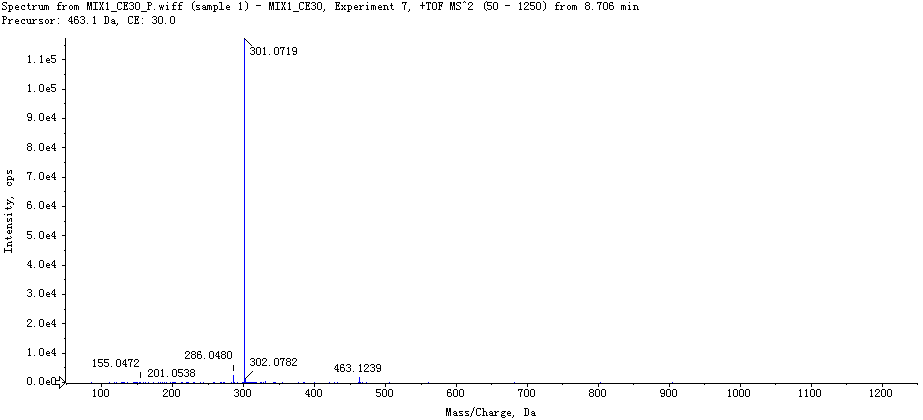

Supplement: Supplementary file 2 [file DataSheet2.zip › Peonidin-3-O-glucoside.png]

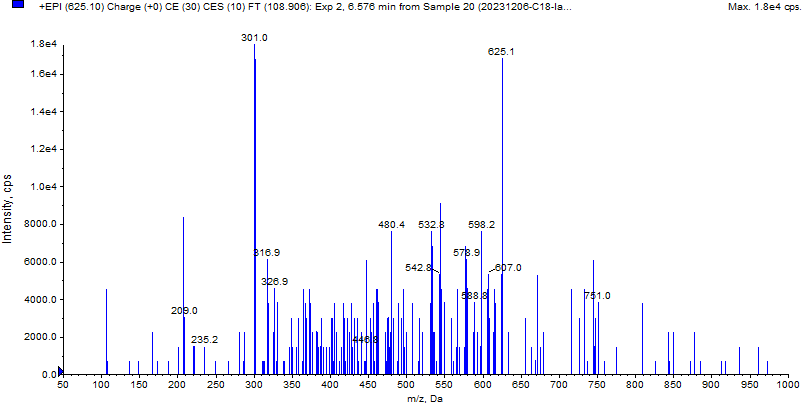

Supplement: Supplementary file 2 [file DataSheet2.zip › Peonidin-3-O-sophoroside.png]

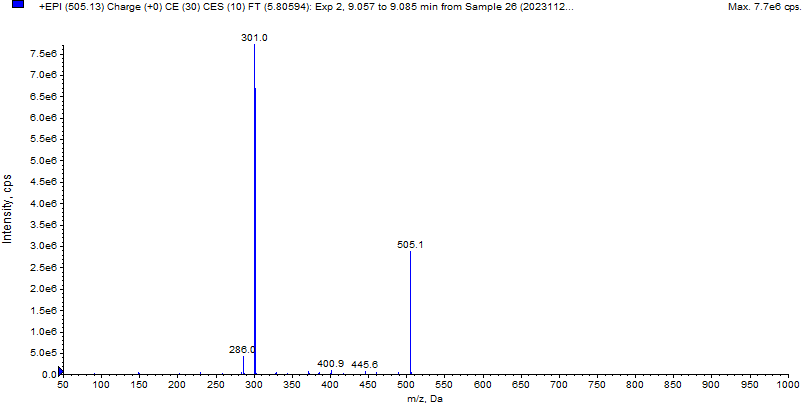

Supplement: Supplementary file 2 [file DataSheet2.zip › Peonidin-3-O-(6''-O-acetyl)galactoside.png]

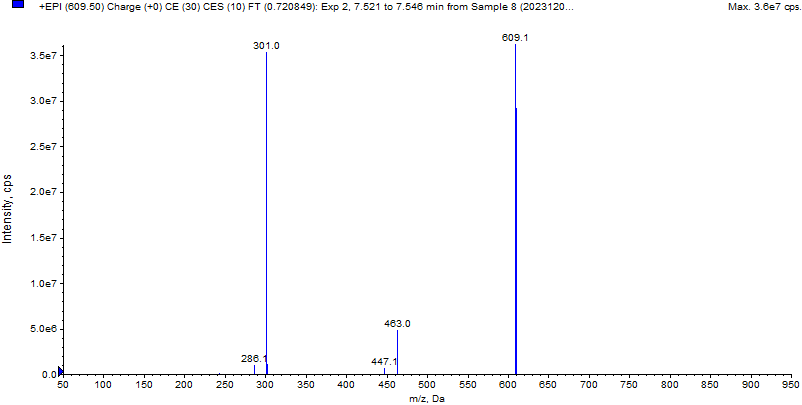

Supplement: Supplementary file 2 [file DataSheet2.zip › Peonidin-3-O-(caffeoyl)rhamnoside.png]

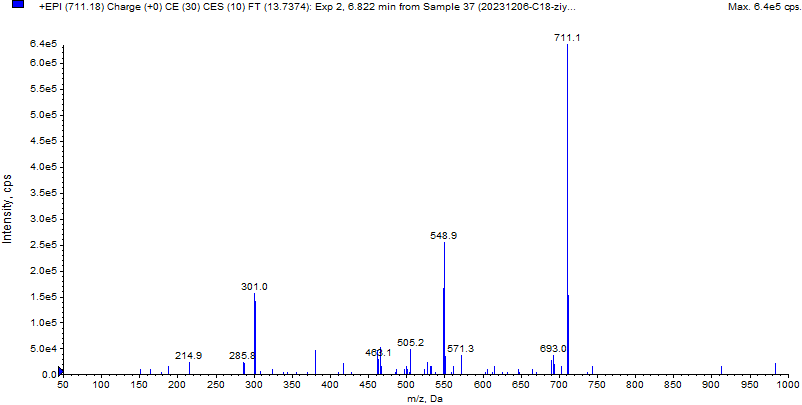

Supplement: Supplementary file 2 [file DataSheet2.zip › Peonidin-3-O-(6''-O-malonyl)diglucoside.png]

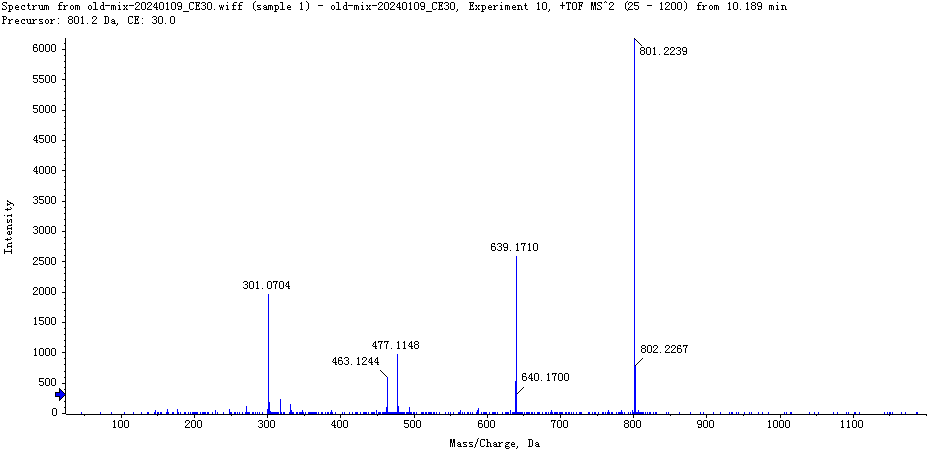

Supplement: Supplementary file 2 [file DataSheet2.zip › Peonidin-sinapoyl-sambubioside.png]

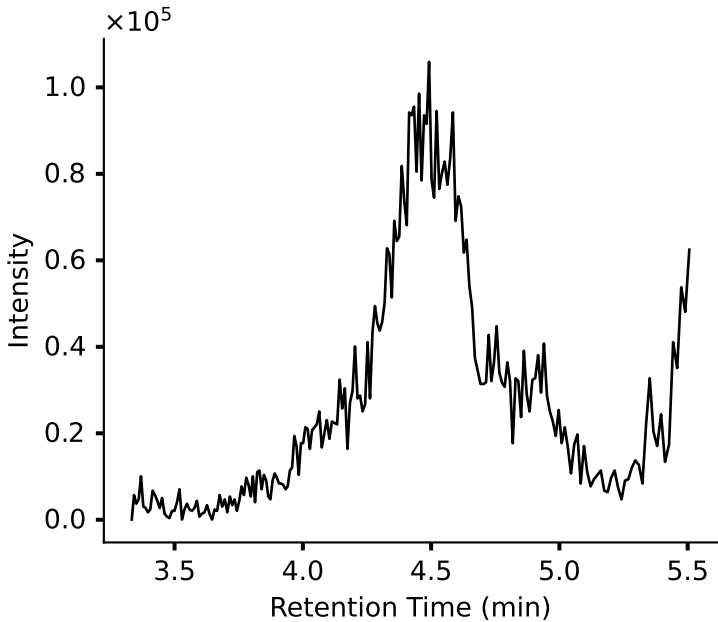

Supplement: Supplementary file 2 [file DataSheet2.zip › Cyanidin-3,5-O-diglucosideXIC.pdf]

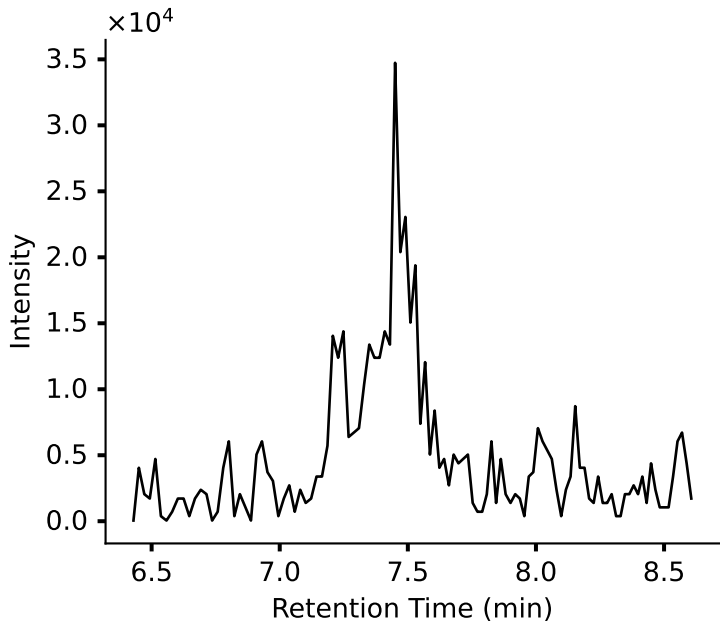

Supplement: Supplementary file 2 [file DataSheet2.zip › Malvidin-3-O-glucosideXIC.pdf]

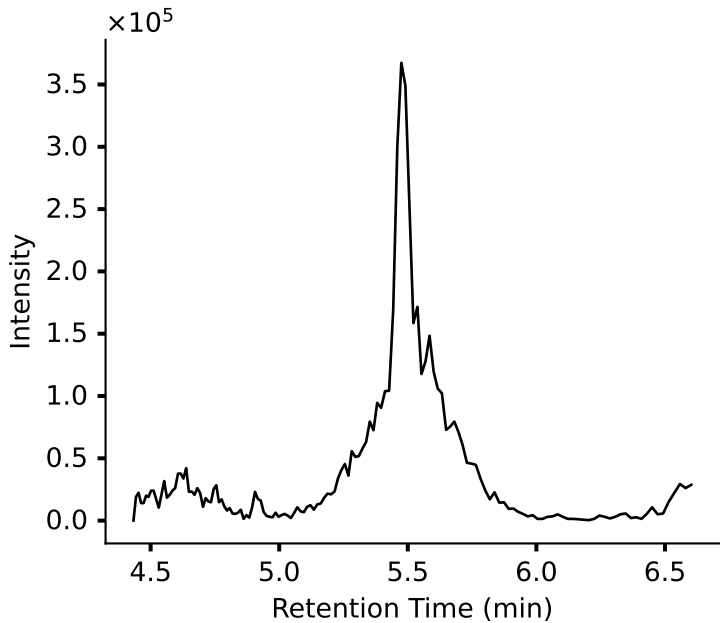

Supplement: Supplementary file 2 [file DataSheet2.zip › Peonidin-3,5-O-diglucosideXIC.pdf]

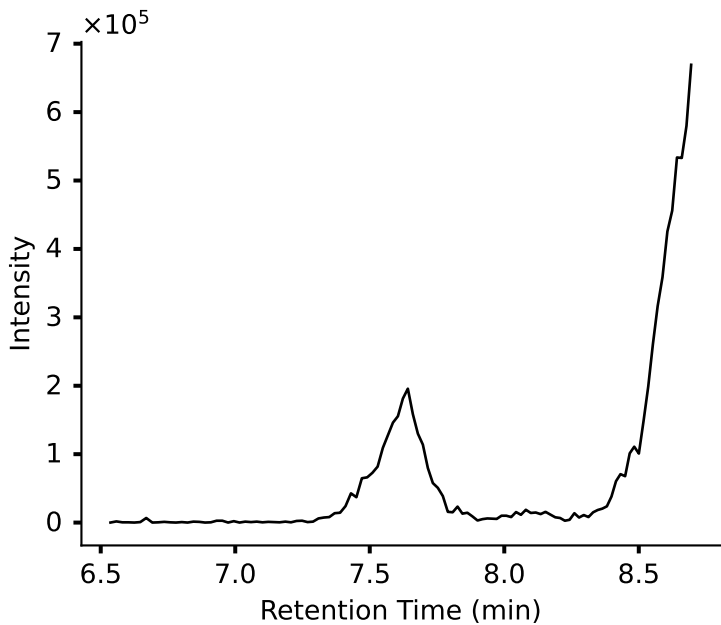

Supplement: Supplementary file 2 [file DataSheet2.zip › Peonidin-3-O-arabinosideXIC.pdf]

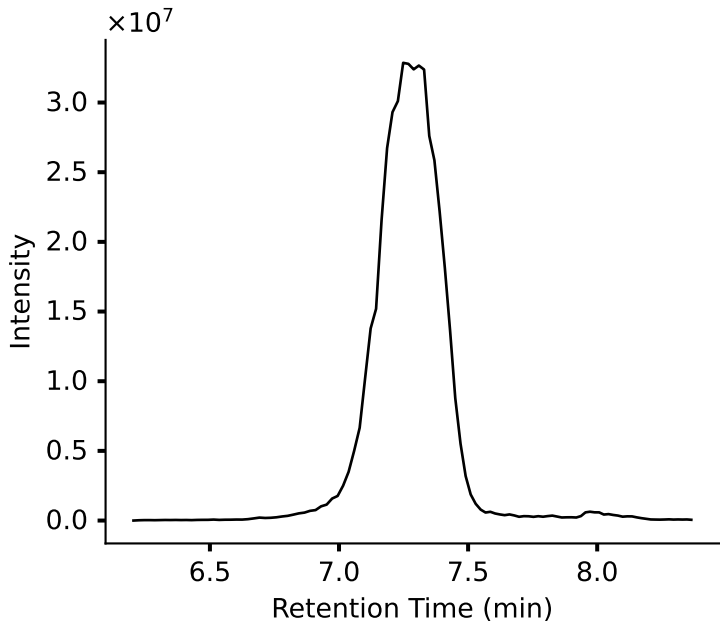

Supplement: Supplementary file 2 [file DataSheet2.zip › Peonidin-3-O-glucosideXIC.pdf]

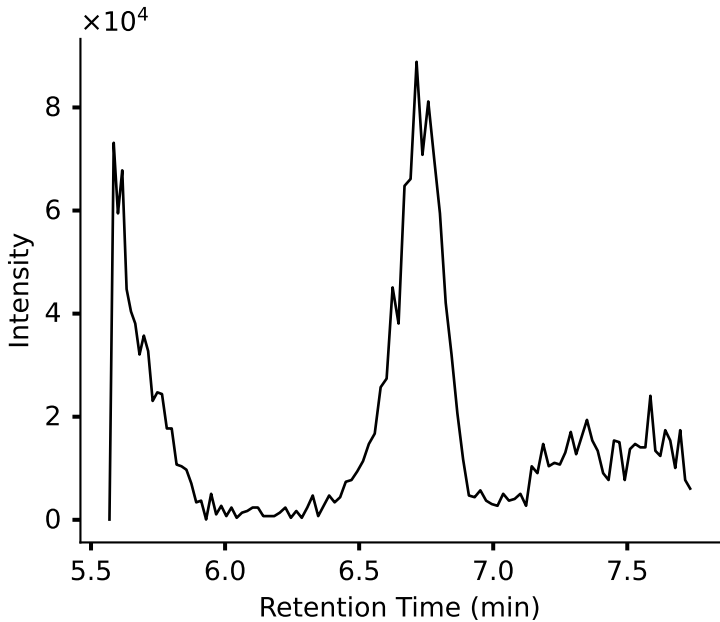

Supplement: Supplementary file 2 [file DataSheet2.zip › Peonidin-3-O-sophorosideXIC.pdf]

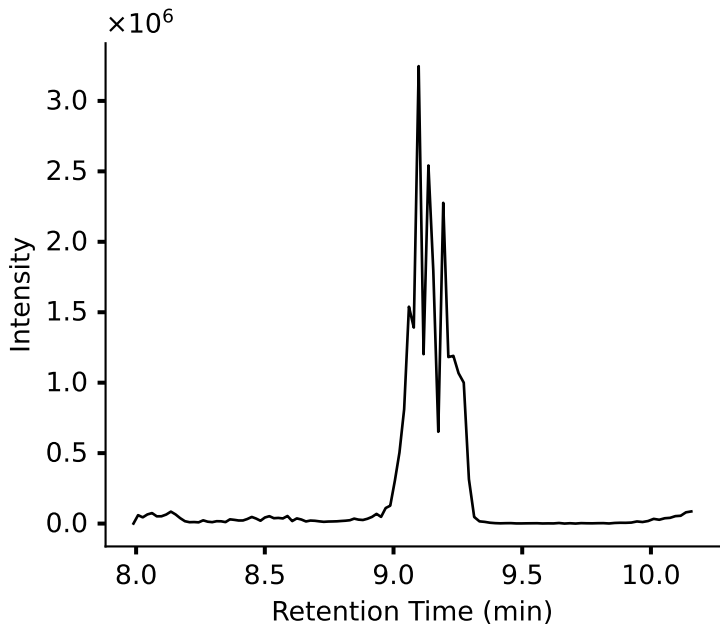

Supplement: Supplementary file 2 [file DataSheet2.zip › Peonidin-3-O-(6''-O-acetyl)galactosideXIC.pdf]

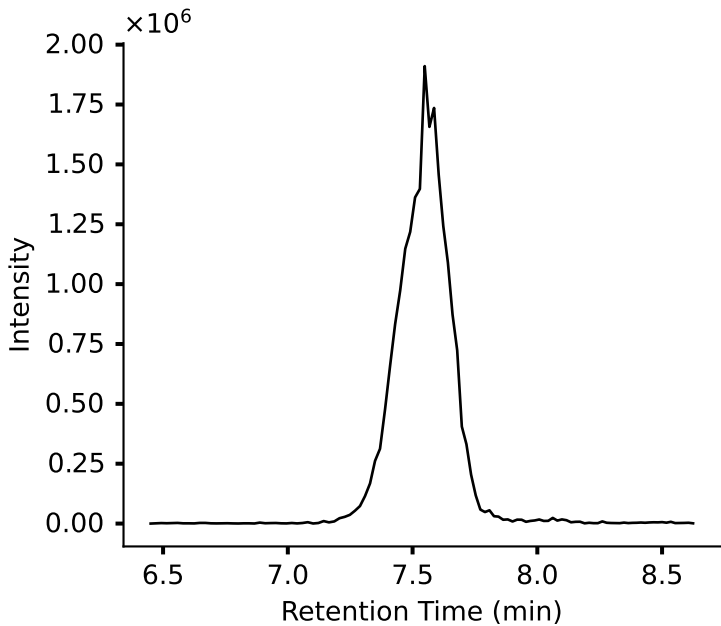

Supplement: Supplementary file 2 [file DataSheet2.zip › Peonidin-3-O-(caffeoyl)rhamnosideXIC.pdf]

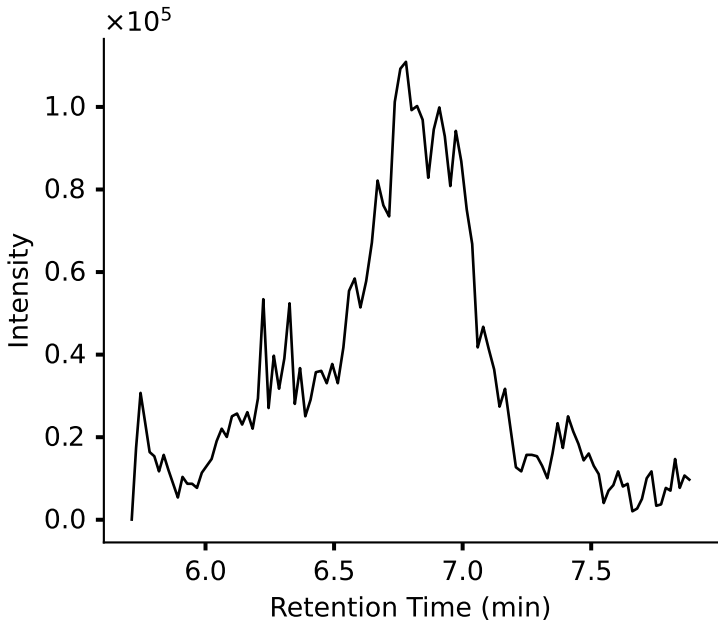

Supplement: Supplementary file 2 [file DataSheet2.zip › Peonidin-3-O-(6''-O-malonyl)diglucosideXIC.pdf]

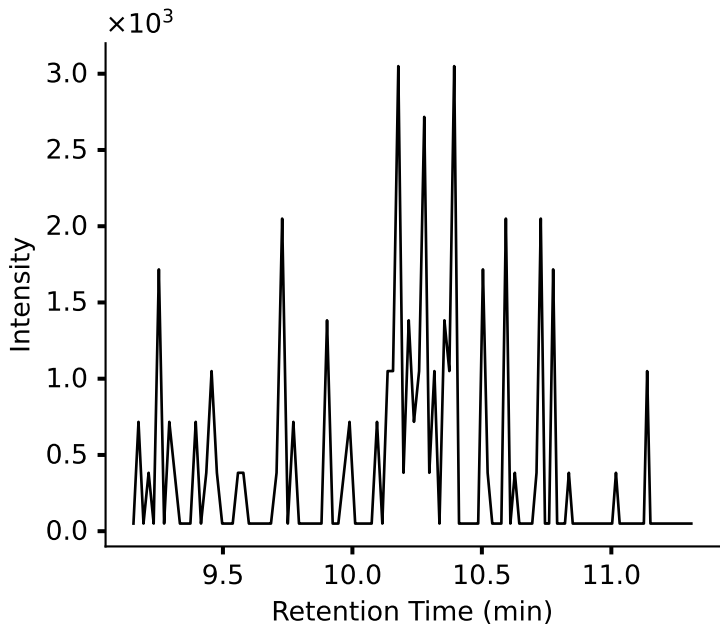

Supplement: Supplementary file 2 [file DataSheet2.zip › Peonidin-sinapoyl-sambubiosideXIC.pdf]

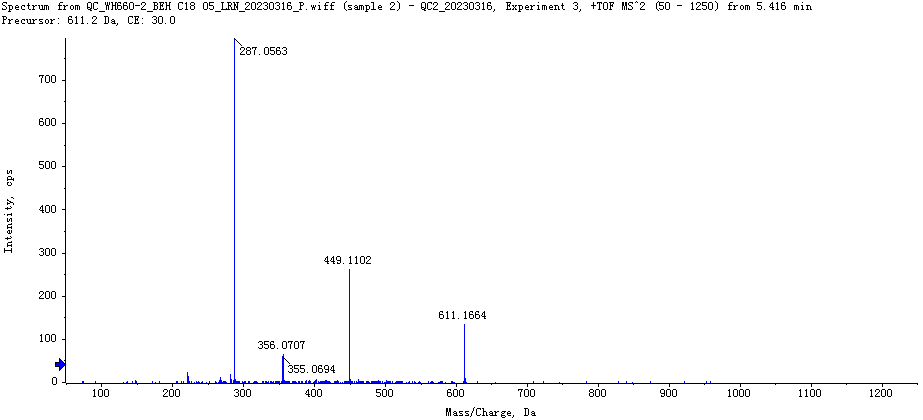

Supplement: Supplementary file 2 [file DataSheet2.zip › Cyanidin-3,5-O-diglucoside.png]

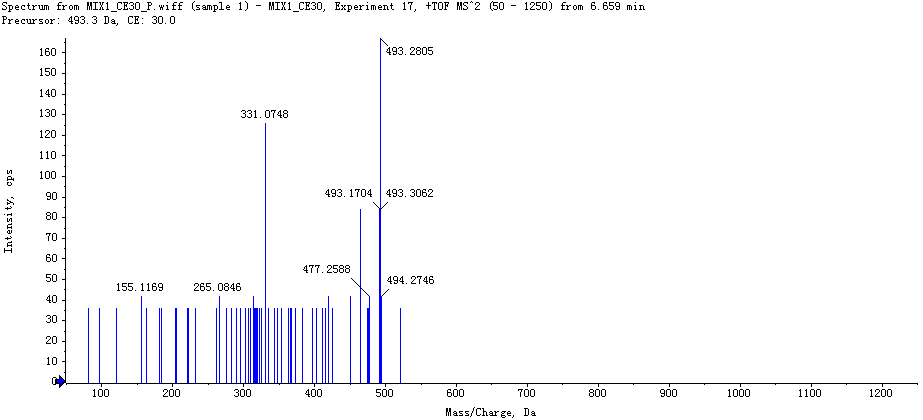

Supplement: Supplementary file 2 [file DataSheet2.zip › Malvidin-3-O-glucoside.png]
